# Airway inflammation and mannitol challenge test in COPD

**DOI:** 10.1186/1465-9921-12-11

**Published:** 2011-01-18

**Authors:** Selma B de Nijs, Niki Fens, Rene Lutter, Erica Dijkers, Frans H Krouwels, Barbara S Smids-Dierdorp, Reindert P van Steenwijk, Peter J Sterk

**Affiliations:** 1 Department of Respiratory Medicine, Academic Medical Centre and University of Amsterdam, Meibergdreef 9, Amsterdam, 1105 AZ, The Netherlands; 2 Department of Experimental Immunology, Academic Medical Centre and University of Amsterdam, Meibergdreef 9, Amsterdam, 1105 AZ, The Netherlands; 3 Department of Pulmonology, Onze Lieve Vrouwe hospital, Oosterpark 9, Amsterdam, 1091 AC, The Netherlands

## Abstract

**Background:**

Eosinophilic airway inflammation has successfully been used to tailor anti-inflammatory therapy in chronic obstructive pulmonary disease (COPD). Airway hyperresponsiveness (AHR) by indirect challenges is associated with airway inflammation. We hypothesized that AHR to inhaled mannitol captures eosinophilia in induced sputum in COPD.

**Methods:**

Twenty-eight patients (age 58 ± 7.8 yr, packyears 40 ± 15.5, post-bronchodilator FEV_1 _77 ± 14.0%predicted, no inhaled steroids ≥4 wks) with mild-moderate COPD (GOLD I-II) completed two randomized visits with hypertonic saline-induced sputum and mannitol challenge (including sputum collection). AHR to mannitol was expressed as response-dose-ratio (RDR) and related to cell counts, ECP, MPO and IL-8 levels in sputum.

**Results:**

There was a positive correlation between RDR to mannitol and eosinophil numbers (r = 0.47, p = 0.03) and level of IL-8 (r = 0.46, p = 0.04) in hypertonic saline-induced sputum. Furthermore, significant correlations were found between RDR and eosinophil numbers (r = 0.71, p = 0.001), level of ECP (r = 0.72, p = 0.001), IL-8 (r = 0.57, p = 0.015) and MPO (r = 0.64, p = 0.007) in sputum collected after mannitol challenge. ROC-curves showed 60% sensitivity and 100% specificity of RDR for >2.5% eosinophils in mannitol-induced sputum.

**Conclusions:**

In mild-moderate COPD mannitol hyperresponsiveness is associated with biomarkers of airway inflammation. The high specificity of mannitol challenge suggests that the test is particularly suitable to exclude eosinophilic airways inflammation, which may facilitate individualized treatment in COPD.

**Trial registration:**

Netherlands Trial Register (NTR): NTR1283

## Introduction

Chronic obstructive pulmonary disease (COPD) is an inflammatory airway disease characterized by non-reversible airflow limitation[1]. Airflow limitation is usually progressive and associated with an abnormal inflammatory response of the lungs to noxious particles or gasses. The treatment options in COPD are still limited and current efforts focus on therapy targeted to particular phenotypes of the disease[1]. A non-invasive, standardised way to measure and monitor airway inflammation in COPD is hypertonic saline-induced sputum[2]. Analysis of induced sputum provides information about cell counts (eosinophils, neutrophils, lymphocytes, macrophages) and cell activity by mediator concentrations (*e.g*. ECP, MPO and IL-8).

In COPD patients the identification of sputum eosinophilia has shown to be of clinical value as it predicts a response to corticosteroids[3-5]. Furthermore, guiding inhaled steroid therapy by sputum eosinophil counts leads to a reduction in exacerbations in COPD, without an increase in steroid dose[6]. These observations demonstrate the value of identifying inflammatory subphenotypes in the treatment of COPD. However, the application of sputum analysis is somewhat limited by the requirement of lab facilities and the not-directly available results. Therefore, there is a need for adequate surrogate markers of airway inflammation in COPD.

Airway hyperresponsiveness (AHR) may serve as a surrogate measure of airway inflammation, since it is associated with the presence of inflammatory cells and release of mediators in the airways[7]. In particular, this holds for indirect challenges, amongst which dry powder mannitol challenge is relatively easy to apply[8,9]. Local mannitol deposition results in an osmotic change, likely to induce the release of mediators from inflammatory cells in the airways[10]. Studies in asthma showed that AHR to mannitol is indeed related to the degree of eosinophilic airway inflammation and is sensitive to treatment with inhaled corticosteroids[11-13]. Interestingly, a proof of concept study demonstrated that mannitol challenge might also be useful in identifying COPD patients who will most likely benefit from inhaled corticosteroids[14]. This may suggest that AHR to mannitol identifies the degree of eosinophilic inflammation in COPD.

We postulated that AHR to mannitol captures eosinophilic airway inflammation in adults with mild to moderate COPD. Our aim was to test this hypothesis by examining the relationship between AHR to mannitol and markers of inflammation in hypertonic saline-induced sputum, blood and exhaled air. As secondary aim, we investigated whether similar relations can be observed when using spontaneously produced sputum during or directly after the mannitol challenge itself. Finally, we constructed receiver operating characteristic (ROC) curves using AHR against sputum eosinophilia in COPD.

## Methods

### Patients

Thirty-two patients with mild to moderately severe COPD were included from two respiratory clinics in Amsterdam, The Netherlands. The definition of COPD was based on GOLD[1]. Inclusion criteria were symptoms of dyspnea, chronic cough or sputum production, current or ex-smoker with at least 20 packyears of smoking history, postbronchodilator FEV_1 _>1.5 liter and >50% of predicted value, FEV_1_/FVC <0.70 and clinically stable for ≥ 4 weeks prior to recruitment. Exclusion criteria were (inhaled) steroid therapy or antibiotic treatment or exacerbation or chest infection ≤ 4 weeks prior to recruitment, treatment with β-blockers, respiratory disease other than COPD including known asthma or allergic rhinitis and contra-indications for challenge testing according to international guidelines[15]. Patients were asked to withhold strenuous exercise and smoking for 6 hrs and eating for 2 hrs; caffeine and short-acting bronchodilators for 8 hrs; long-acting bronchodilators for 48 hrs; short-acting anti-cholinergics for 24 hrs; long-acting anti-cholinergics and anti-histamines for 72 hrs; and leukotriene antagonists for 4 days prior to the mannitol challenge.

The study was approved by the Hospital Medical Ethics Committee and all patients gave their written informed consent. The study was registered in the Netherlands trial register under NTR 1283, was designed, performed and analysed by the authors, and was not sponsored by others than the Academic Medical Centre, Amsterdam, The Netherlands itself.

### Study design

**Figure 1:**
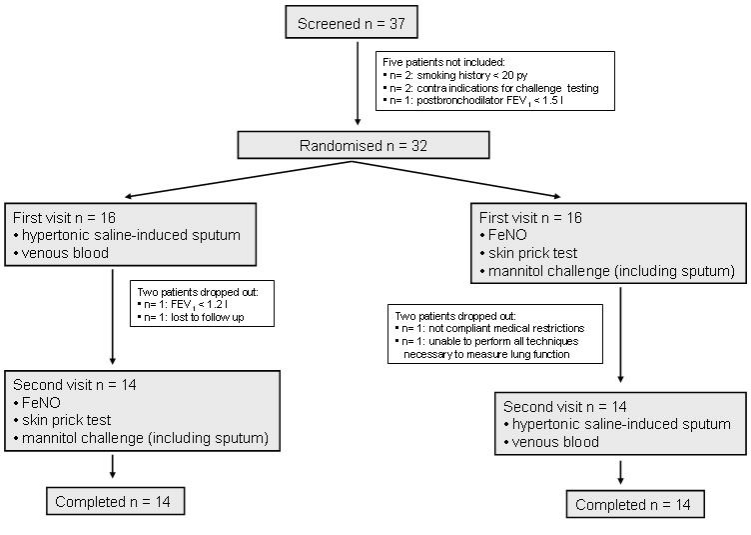
**Study design**.

The study had a cross-sectional design with two studies days comprising randomized challenges with hypertonic saline and mannitol (figure 1). At a separate screening visit, inclusion and exclusion criteria were examined, postbronchodilator (400 μg salbutamol) spirometry was performed and diffusion capacity was measured.

The sequence of the two study visits was randomized [interval (median (range)):7(7-15) days]. On one day sputum was induced by hypertonic saline and a venous blood sample was obtained. On the other day exhaled nitric oxide was measured first, followed by assessment of atopy and mannitol challenge testing.

### Measurements

#### Lung function

Spirometry (MasterscreenPneumo; Jaeger; Würzburg, Germany) was performed by a trained respiratory technician according to the latest recommendations[16]. Diffusion capacity of the lung for carbon monoxide (D_L, CO_) was measured according to the recommendations using the single breath method and was corrected for haemoglobin[17].

#### Mannitol challenge

Mannitol challenge was performed using a commercially available kit (Pharmaxis Ltd; Sydney, Australia) as described by Anderson et al[8]. Patients inhaled sequential doses of 5, 10, 20, 40, 80, 160, 160 and 160 mg of mannitol via the inhaler. The test stopped when 15% fall in FEV_1 _was achieved or the cumulative dose of 635 mg had been administered. Response-dose-ratio (RDR) was calculated as the%fall in FEV_1 _at the last dose, divided by the total cumulative dose mannitol (%fall.mg) in milligrams administered[18].

If a patient had to cough spontaneously during the mannitol challenge, he or she was asked to expectorate. This sputum was labeled as mannitol-induced sputum.

#### Sputum induction and processing

Prior to sputum induction, patients inhaled 200 μg salbutamol. Sputum was induced by inhalation of NaCl 4.5% during 3 × 5 min intervals[19]. This sputum was labeled as induced sputum.

Whole sputum samples were processed according to a protocol that has been validated in our laboratory[20]. Differential cell counts were expressed as the percentage of non-squamous cells. Absolute cell numbers were calculated as (% cell × total cell count)/sputum weight. Sputum samples containing >80% non-squamous cells were excluded from analysis.

All sputum cell counts were performed by one experienced and qualified technician blinded to the clinical details. As an extra control 10% of the samples were analyzed by a second technician.

#### Analysis of soluble markers in sputum supernatant

Levels of eosinophil cationic protein (ECP; detection limit >60 pg/ml), myeloperoxidase (MPO; detection limit >1.5 ng/ml), interleukin-8 (IL-8; detection limit >19.1 pg/ml) and alpha-2-macroglobulin (α_2_M; detection limit >2.1 mg/ml) were measured by enzyme-linked immunosorbent assays (ELISA)[21,22].

#### Exhaled Nitric Oxide (FeNO)

FeNO was measured with a portable rapid-response chemoluminescent analyser (flow rate 50 mL/s; NIOX System, Aerocrine, Sweden) according to recent guidelines[23].

#### Statistical analysis

The relationship between AHR to mannitol (RDR) and the markers of airway inflammation were analyzed using Pearson's correlation coefficient (r_p_). Non-normally distributed data were log-transformed for further analysis. If no cells were counted, a value of 0.1 was taken before log-transformation. Receiver operating characteristic (ROC) curves were constructed, using RDR against eosinophilic vs non eosinophilic COPD (threshold 2.5% sputum eosinophils). Wilcoxon signed rank test and Bland-Altman analysis was used to compare cell counts of the two sputum samples.

A sample size estimation showed that the detectable value of the correlation (r) under the alternative hypothesis with a sample of 23 patients (*n*) is between 1-0.55 (power = 0.808; alpha = 0.05). Therefore, thirty-two patients were recruited taking into account an expected 10% drop-out rate and a 20% probability of missing or non-valid data.

## Results

Twenty-eight of the 32 patients completed the study (table 1). Four patients dropped out for reasons of: non-compliance with medication restrictions (n = 1), lost to follow up (n = 1), FEV_1 _<1.2 litre prior to challenge (n = 1) and inability to perform all techniques necessary to measure lung function (n = 1). Two out of 28 mannitol challenges were not completed for reasons of coughing (n = 1) and tiredness (n = 1), but these patients were included since this was not an exclusion criterion. Hypertonic saline-induced sputum was collected in 28 patients and mannitol-induced sputum in 21 patients.

**Table 1:** Patient characteristics I

	*n *= 28
Male/Female (*n*)	23/5
Gold I/II (*n*)	12/16
Age (years)	58 ± 7.8
Current/ex-smoker (*n*)	12/16
Smoking history (pack years)	40 ± 15.5
Inhaled corticosteroids before study (*n*)	14
Postbronchodilator FEV_1 _(L)	2.57 ± 0.6
Postbronchodilator FEV_1 _(%predicted)	77 ± 14.0
FEV_1_/FVC	0.55 ± 0.08
Atopy (*n*)	3
D_L,CO _(% predicted)	65 ± 14.7

Values are expressed as mean ± SD

Gold, Global Initiative for Chronic Obstructive Lung Disease.; FEV_1_, forced expiratory volume in 1 s; FVC, forced vital capacity.; DLCO, Diffusion capacity lung for carbon monoxide

### Correlation of inflammatory markers in hypertonic saline-induced sputum and blood with AHR to mannitol

**Figure 2:**
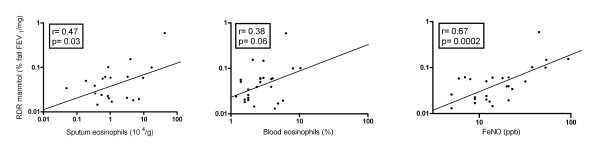
**Correlation AHR to mannitol and eosinophils in *hypertonic saline-induced *sputum (left), blood eosinophils (middle) and fraction exhaled nitric oxide (right)**.

The baseline values for airway hyperresponsiveness and inflammatory markers are presented in table 2. Five hypertonic saline-induced sputum samples were excluded from analyses as a result of >80% non-squamous cells on differential cell counts. There was a significant positive correlation between the degree of AHR to mannitol (RDR mannitol) and eosinophil counts (r = 0.47, p = 0.03, figure 2) per gram hypertonic saline-induced sputum and with IL-8 levels (r = 0.46, p = 0.04). The correlation between RDR mannitol and blood eosinophils was borderline significant (r = 0.38, p = 0.06, figure 2). No other correlations between RDR mannitol and hypertonic saline-induced sputum parameters were found (Table 3). In addition, a significant, positive association between RDR mannitol and the level of FeNO (r = 0.67, p = 0.0002, figure 2) was observed. When using PD_15 _to mannitol, the correlation coefficients with sputum and blood eosinophils counts were -0.38 (p = 0.09) and -0.43 (p = 0.03), respectively.

**Table 2:** Patient characteristics II- Airway hyperresponsiveness (AHR) and airway inflammation

Subjects	*n *= 28
Airway responsiveness (*n*= 26)	
- AHR to mannitol*(*n*)	18
- RDR mannitol (%/mg)	0.044 (0.0204-0.0605)
- Max dose of mannitol (mg)	395 (315-635)
- PD_15 _mannitol**	331 (196-635)
Fraction Exhaled Nitric Oxide	
- FeNO (ppb)	14 (9-22.5)
Sputum (*n*= 23)	
- Eosinophils (%)	0.8 (0.4-3.1)
- Lymphocytes (%)	1.4 (1.0-2.4)
- Macrophages (%)	18.8 (12.8-22.2)
- Neutrophils (%)	77.2 (70.8-86.0)
- Total cell count (x10^6^/g)	1.6 (0.5-2.8)
Blood (*n*= 28)	
- Eosinophils (%)	2.7 (1.8-4.3)
- Neutrophils (%)	55.9 (49.8-61.8)

Values are expressed as median and interquartile range

RDR, Response Dose Ratio (fall FEV_1 _divided by cumulative dose given); PD_15, _Provocation Dose of mannitol to cause a 15% fall in FEV_1_; pbb, parts per billion.

*: positive reaction to mannitol: PD_15 _<635 mg; **: including 8 patients who did not reach a PD_15_, we used an assigned value of 635 mg

**Table 3:** Correlation between AHR to mannitol expressed by the response-dose ratio (RDR) and markers of airway inflammation

	r	p- value
FEV_1 _(% predicted)	-0.09	0.67
Log FeNO	0.67	0.0002*
*Hypertonic saline-induced sputum*		
Log (10^4^/g) eosinophils	0.47	0.03*
Log (10^4^/g) lymphocytes	0.18	0.45
Log (10^4^/g) macrophages	0.27	0.24
Log (10^4^/g) neutrophils	0.25	0.28
Log (10^4^/g) epithelial cells	0.38	0.10
Log (ng/ml) ECP	0.39	0.09
Log (pg/ml) IL-8	0.46	0.04*
Log (ng/ml) MPO	0.33	0.14
*Mannitol- induced sputum*		
Log (10^4^/g) eosinophils	0.71	0.001*
Log (ng/ml) ECP	0.72	0.001*
Log (pg/ml) IL-8	0.57	0.015*
Log (ng/ml) MPO	0.64	0.007*
*Venous blood*		
Log (%) eosinophils	0.38	0.06
Log (%) neutrophils	-0.23	0.26

RDR is taken as the maximal% fall in FEV_1 _per cumulative dose; correlation: Pearsons correlation coefficient; FeNO: fraction exhaled nitric oxide in parts per billion. * p < 0.05

### Mannitol- induced sputum markers

**Figure 3:**
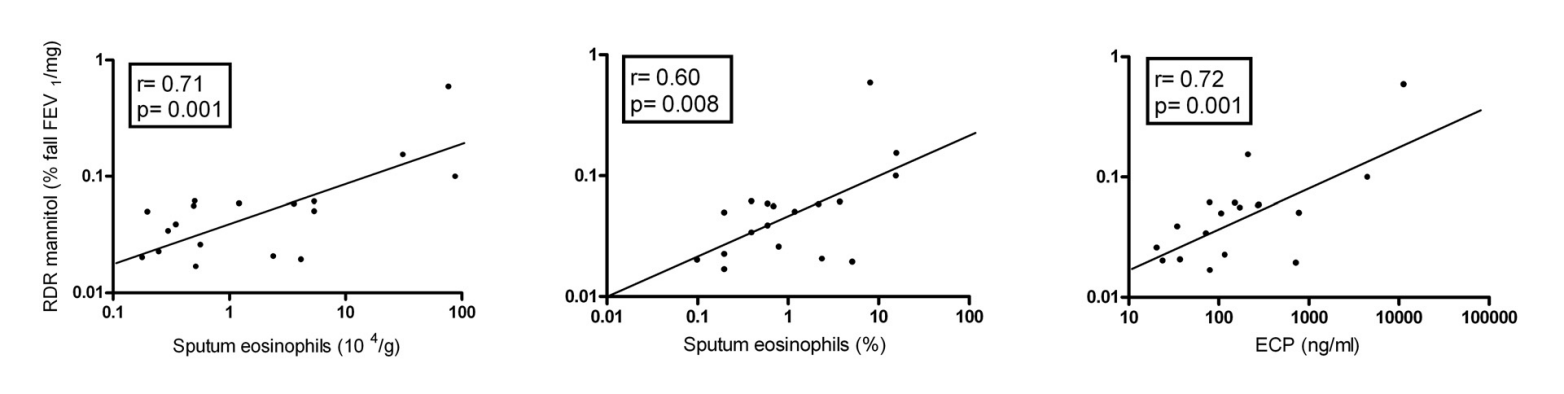
**Correlation AHR to mannitol and the absolute (left) and relative (middle) amount of eosinophils and ECP (right) in *mannitol- induced *sputum**.

Two out of 21 sputum samples were excluded from analyses as a result of >80% non-squamous cells on differential cell counts. There were strongly significant positive correlations between RDR mannitol and the absolute and relative numbers of eosinophils and the level of ECP in mannitol-induced sputum (r = 0.71, p = 0.001; r = 0.60, p = 0.008; r = 0.72, p = 0.001, respectively) (Figure 3). In addition, RDR mannitol was related to the levels IL-8 (r = 0.57, p = 0.015) and MPO (r = 0.64, p = 0.007) (Table 3).

Inflammatory markers as obtained by hypertonic- and mannitol challenge were generally well correlated (Table 4). The limits of agreement by Bland and Altman analyses for eosinophil counts and log ECP were -5.7-8.6% and -0.73-0.72, respectively.

**Table 4:** Induced sputum total and differential cell count and mediators when collected with hypertonic saline or mannitol (18 paired samples)

	Hypertonic saline	Mannitol induced	r
Eosinophils (10^4^/g)	1.1 (0.5-6.5)	0.9 (0.3-7.5)	0.81 (p = < 0.001*)
Lymphocytes (10^4^/g)	1.8 (1.0-7.0)	1.9 (1.0-3.9)	0.37 (p= 0.132)
Macrophages (10^4^/g)	31.7 (13.2-48.7)	20.5 (12.6-34.4)**	0.71 (p = 0.001*)
Neutrophils (10^4^/g)	111.1 (59.4-231.5)	108.1 (58.4-160.1)	0.75 (p = < 0.001*)
Epithelial cells (10^4^/g )	21.3 (14.5-71.2)	25.5 (12.8-42.3)	0.61 (p = 0.009*)
Total cell count (×10^6^/g)	1.6 (0.8-2.6)	1.4 (0.8-2.0)	0.73 (p = 0.001*)
Gram sputum	6.9 (5.2-10.9)	4.0 (2.0-9.0)**	0.60 (p = 0.006*)
ECP (ng/ml)	147.5 (89.5-492.3)	125.8 (64.1-277.2)	0.85 (p = < 0.001*)
IL-8 (pg/ml)	1925.5 (534.8-7076.0)	1595 (862.8-3357.2)	0.72 (p = 0.001*)
MPO (ng/ml)	4529.7(1779.4-7414.8)	5174.3(1203.0-11933)	0.84 (p = < 0.001*)

Data expressed as median and interquartile range; r= Pearsons correlation coefficient:

* = significant; **= significantly different (Wilcoxon rank test).

### ROC curves

**Figure 4:**
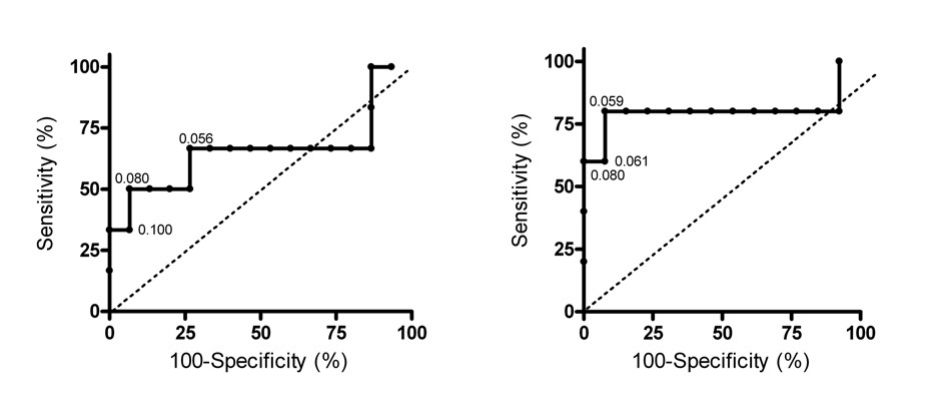
**ROC curve**. The curve of sensitivity against 100-specificity is based on using reactivity to mannitol, given as RDR values (%fall.mg), to predict eosinophilic COPD (>2.5%) in *hypertonic saline *(left) and *mannitol-induced *(right) sputum. Dotted line: line of identity.

The overall accuracy of RDR to mannitol for the assessment of eosinophilic or non eosinophilic COPD, described as the area under the ROC curve (Figure 4), was 67% (95% CI, 33.6 to 97.5%) for hypertonic saline- and 80% (95% CI, 47.7 to 112.3%) for mannitol-induced sputum. At RDR of 0.08%fall.mg the sensitivity and specificity for >2.5% eosinophils in hypertonic saline-induced sputum was 50% (95% CI, 11.8 to 88.2%) and 93% (95% CI, 68 to 99.8%), respectively. For mannitol-induced sputum the sensitivity and specificity was 60% (95% CI, 14.7 to 94.7%) and 100% (95% CI, 75.3 to 100%) respectively (Figure 4). When using a cut-point of 2.0% eosinophils we observed sensitivities of 44% (95% CI, 13.7 to 78.8%) and 43% (95% CI, 9.0 to 81.6%) with specificities of 100% (95% CI, 73.5 to 100.0%) and 100% (95% CI, 71.5 to 100%) for hypertonic saline- and mannitol- induced sputum, respectively.

## Discussion

In this group of mild to moderate COPD patients, AHR to inhaled mannitol was consistently associated with eosinophil counts in hypertonic saline- as well as mannitol-induced sputum. In addition, we observed associations between AHR to mannitol and soluble markers of inflammation in sputum. Our results suggest that mannitol challenge identifies inflammatory subphenotypes in COPD, in particular those patients without eosinophilic inflammation due to the high specificity of the test. This may facilitate individualized treatment in COPD.

To our knowledge, this is the first study assessing the relationship between airway hyperresponsiveness to inhaled mannitol and markers of airway inflammation in sputum and exhaled air in patients with COPD. These observations extend previous findings in COPD using adenosine 5'-monophosphate (AMP), in which a correlation between AHR to AMP and eosinophils in sputum was observed[24]. Interestingly, a similar correlation between RDR to mannitol and sputum eosinophils was recently reported in patients with asthma, also showing absence of eosinophilia in patients without mannitol hyperresponsiveness[13]. Hence, mannitol challenge appears to provide valuable information on the inflammatory profile in both patients with COPD and asthma.

In our study, particular attention was paid to methodological aspects such as selection of COPD patients, design and methods. The patients were derived from a clinical population rather than an epidemiological one, in order to strengthen the applicability of our findings. All patients were well characterised by using subjective and objective criteria. This included the presence of symptoms, fixed airway obstruction and smoking history. The full range in sputum eosinophils counts was 0.1 to 7.4%, which is similar to previous studies in COPD[3-5]. To exclude any confounding effects of inhaled corticosteroids on mannitol challenge, the patients who used inhaled corticosteroids stopped this medication for 4 weeks[9,11]. In order to answer the research question accurately, we performed mannitol challenge and sputum induction on separate days. In addition, we examined sputum expectorated after the mannitol itself, which confirmed our results. Furthermore, as inflammatory markers we used both, the presence of inflammatory cells and markers of cell activation. This provided consistent associations.

Nevertheless, our study has limitations. First, we could not obtain adequate sputum samples in all patients at all time points. Even though the power of the study was adequate to address the primary objectives, it may not have been adequate to examine our secondary objective. Second, we can not exclude that our COPD group included patients who also had asthma. We excluded those with a previous history of asthma, but this may not have sufficed. However, all patients had a smoking history, fixed airflow limitation, met the COPD GOLD criteria, and were diagnosed and treated as COPD patients. Third, the patients needed to stop the inhaled corticosteroids in order to examine unbiased disease markers. Therefore, the test performance cannot be generalized to COPD patients on inhaled steroids. This will require a separate study. Finally, we did not include a second mannitol challenge for examining reproducibility of our results, which is a limitation of our design.

How can we interpret these results? Mannitol is an osmotic stimulus that causes airway narrowing by release of bronchoconstrictor mediators such as leukotrienes, prostaglandins and histamine[25,26]. The source of these mediators is likely to be mast cells and eosinophils in the airways as both these cell types release mediators *in vitro *in response to mannitol[10,11,27]. Mast cells and eosinophils are not unimportant in COPD and may contribute to the fluctuations of airways obstruction as observed e.g. during exacerbations[28-31]. We did not observe associations of mannitol responsiveness with neutrophil counts in sputum or blood, but did found significant correlations with sputum IL-8 and MPO. This may suggest that epithelial cell and neutrophil activity are also involved in determining the airway narrowing to inhaled mannitol in COPD. Interestingly, mannitol responsiveness was more strongly associated with FeNO than with sputum eosinophils. However, we did not find a significant association between the latter two parameters. This is in keeping with the data by Siva *et al*. [6]. Our results suggest that mannitol responsiveness is a better marker of eosinophilic inflammation than FeNO in COPD.

Notably, we observed that most COPD patients produced adequate sputum samples during the mannitol challenge. This occurred even in absence of encouraging the patients to expectorate. Therefore, the success rate of obtaining mannitol-induced sputum may well be improved by adjusting the standard operating procedure of the test. Our findings extend a recent study in asthma, showing adequate sputum samples after mannitol challenge[32]. Inhaled mannitol changes osmolarity and reduces viscoelasticity, surface tension, contact angle and the solids content of sputum[33]. This may explain why 75% of the patients gave up sputum during mannitol challenge. Our results suggest that mannitol activated eosinophils, neutrophils and epithelial cells. Hence, even though AHR to mannitol was associated with eosinophilic airway inflammation, it is likely to be a more pleiotropic stimulus within the airways.

What are the clinical implications of our study? Eosinophilic airway inflammation predicts the response of COPD patients to systemic and inhaled corticosteroids[4,5]. In addition, inhaled steroid therapy guided by sputum eosinophils reduces exacerbation rate in patients with COPD[6]. Our results suggest that mannitol challenge can identify COPD patients without eosinophilic airway inflammation, who not likely to benefit from inhaled steroid therapy[6]. This subphenotype of patients cannot be distinguished from other patients with COPD on clinical grounds or lung function criteria. Therefore, mannitol challenge may qualify as a feasible alternative in the monitoring of anti-inflammatory therapy in COPD. The high specificity (100%) in combination with limited sensitivity indicates that mannitol responsiveness is particularly suitable to exclude sputum eosinophilia in COPD. Indeed, inhaled steroids appear to be ineffective in COPD patients with the lowest responsiveness to mannitol[14]. Therefore, mannitol responsiveness may support decisions to refrain from inhaled steroid treatment, thereby potentially preventing overtreatment of COPD. This requires a randomized controlled study in COPD comparing a treatment strategy based on AHR to mannitol with the currently recommended treatment strategy based on clinical markers only. It remains to be established whether mannitol challenge can also be an outcome measure of the efficacy of steroids in COPD, as has been shown in asthma[12]. Finally, our data suggest that the assessment of AHR and airway inflammation in COPD can be combined in a single test. This would have large practical advantages, not only in clinical research, but also regarding the guidance and monitoring of anti-inflammatory therapy in clinical practice.

## Conclusions

We conclude that airway responsiveness to mannitol can be used to rule out eosinophilic airway inflammation in patients with mild to moderate COPD who are not treated with inhaled corticosteroids. These finding suggests that mannitol challenge is a candidate for the guidance and monitoring of individualized, anti-inflammatory therapy in COPD, as an alternative to sputum eosinophils.

## List of abbreviations

AHR: airway hyperresponsiveness; ECP: eosinophil cationic protein; FeNO: fraction exhaled nitric oxide; FEV_1_: forced expiratory volume in one second; FEV_1_/FVC: forced vital capacity divided by the forced expiratory volume in one second; FVC: forced vital capacity; ICS: inhaled corticosteroids; IL-8: interleukin-8; MPO: myeloperoxidase; PD_15_: provocation dose to cause a fall in FEV_1 _>15%; RDR: response dose ratio; ROC: receiver operating characteristic.

## Competing interests

The authors declare that they have no competing interests.

## Authors' contributions

SdN was the main author of the paper and developed the study design and subject recruitment, collected study data and performed statistical analysis. All other authors contributed significantly to the design of the study, the collection and assessment of clinical data and development of this paper. All authors contributed significantly to the development of the manuscript and all have seen and approved the final version and take responsibility for the content.
